# Supplementary material for: Endo-lysosomal Aβ concentration and pH trigger formation of Aβ oligomers that potently induce Tau missorting
Source: Nat Commun. 2021 Jul 30;12:4634. doi: 10.1038/s41467-021-24900-4 (PMC8324842; doi:10.1038/s41467-021-24900-4)
Supplement: Supplementary file 1 — Supplementary Information [file 41467_2021_24900_MOESM1_ESM.pdf]

Endo-lysosomal Aβ concentration and pH trigger formation of Aβ oligomers that potently induce Tau missorting

Marie P. Schützmann[1,†], Filip Hasecke[1,†], Sarah Bachmann[2,†], Mara Zielinski[3], Sebastian Hänsch[4], Gunnar F. Schröder[3,5], Hans Zempel[2,*], and Wolfgang Hoyer[1,3,*]

[1]Institut für Physikalische Biologie, Heinrich-Heine-Universität Düsseldorf, 40204 Düsseldorf, Germany

[2]Institute of Human Genetics and Center for Molecular Medicine Cologne (CMMC), University of Cologne, Faculty of Medicine and University Hospital Cologne, 50931 Cologne, Germany

[3]Institute of Biological Information Processing (IBI-7) and JuStruct: Jülich Center for Structural Biology, Forschungszentrum Jülich, 52425 Jülich, Germany

[4]Department of Biology, Center for Advanced Imaging (CAi), Heinrich-Heine-Universität Düsseldorf, 40204 Düsseldorf, Germany

[5]Physics Department, Heinrich-Heine-Universität Düsseldorf, 40204 Düsseldorf, Germany

Correspondence and requests for materials should be addressed to H.Z. (email: hans.zempel@uk-koeln.de) or W.H. (email: wolfgang.hoyer@hhu.de)

[†] These authors contributed equally.

Supplementary Figures

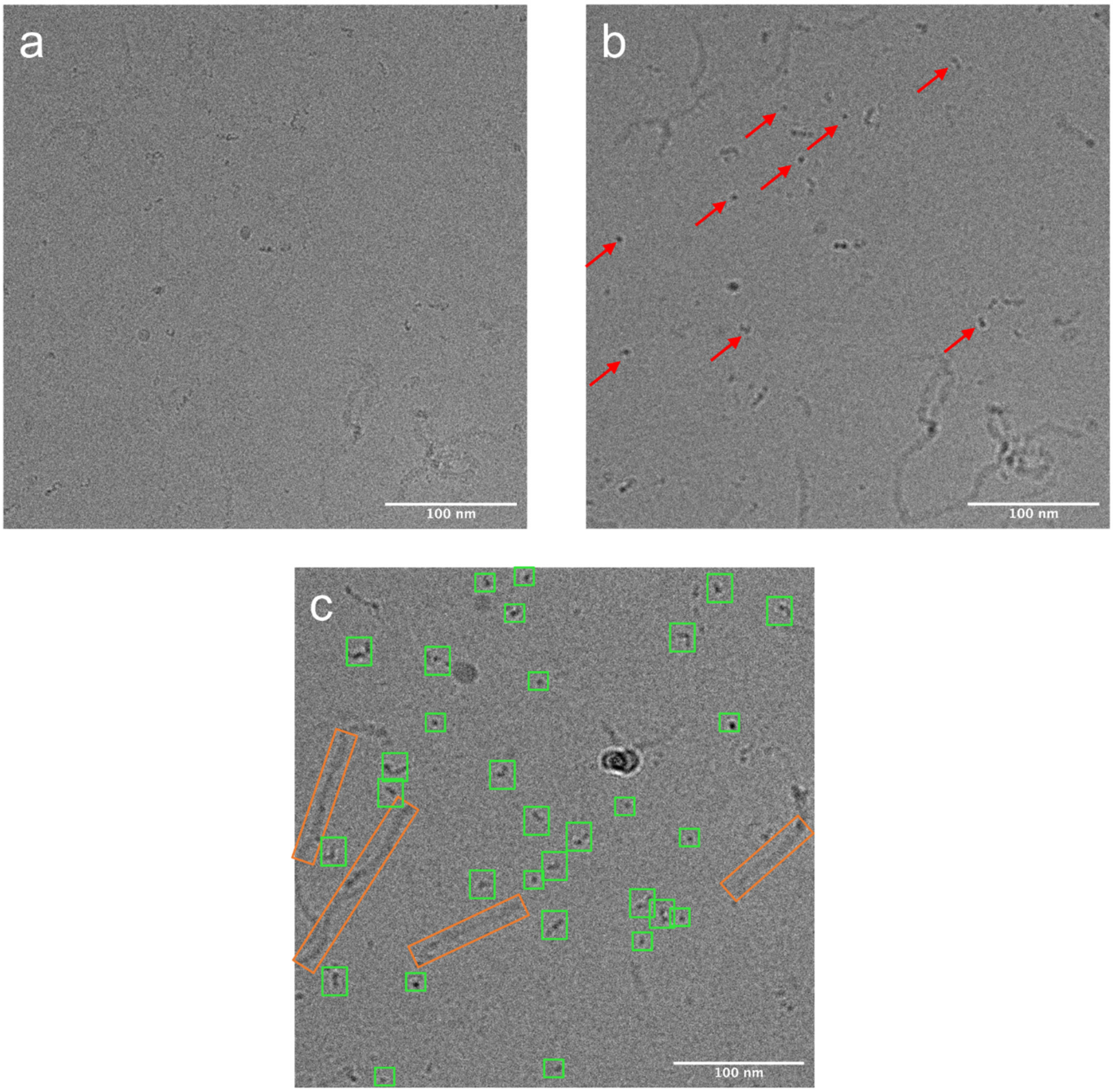

Supplementary Fig. 1 Representative examples from a total of 1308 cryo-EM micrographs collected of dimAβ AβOs. Micrographs were recorded at a defocus of **a** -1.6 µm or **b** -6 µm, respectively. Small AβO particles, indicated by red arrows, were selected on the high defocus micrographs for density reconstruction. **c** Thirty micrographs were analyzed to estimate the relative abundance of the small AβOs (green boxes) and AβO protofibrils (orange boxes). All clearly discernible objects were counted. Small AβOs accounted for 72±12% of all particles. Considering the particles' dimensions, we estimated that small AβOs contain 2-3% of all Aβ molecules within AβOs.

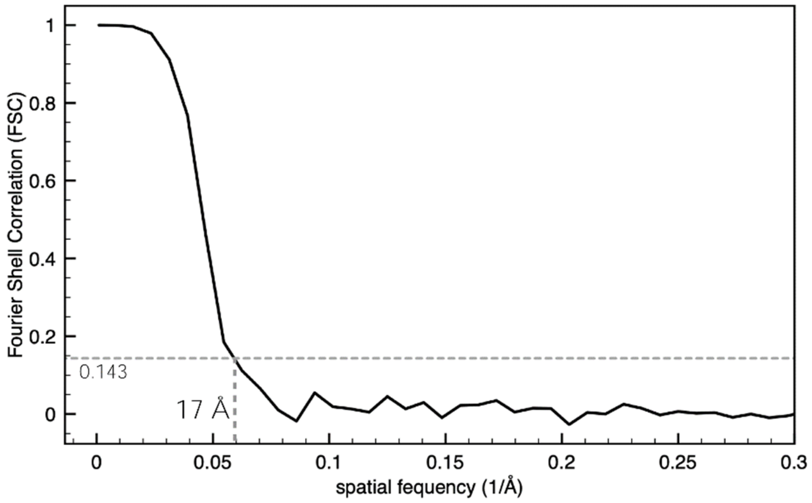

Supplementary Fig. 2 Fourier shell correlation (FSC) for the 3D reconstruction of the smallest dimAβ AβOs observed on the cryo-EM micrographs yields a resolution estimate of 17 Å.

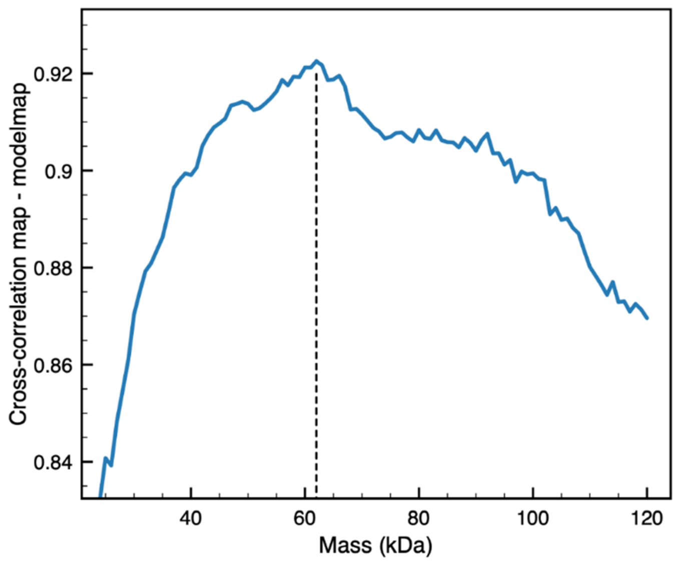

Supplementary Fig. 3 Density cross-correlation computed for each of the 110 pseudo-atomic model maps with the EM reconstruction after sharpening with VISDEM using the corresponding mass of the pseudo-atomic model. The highest correlation (0.923) is obtained for a mass of 62 kDa.

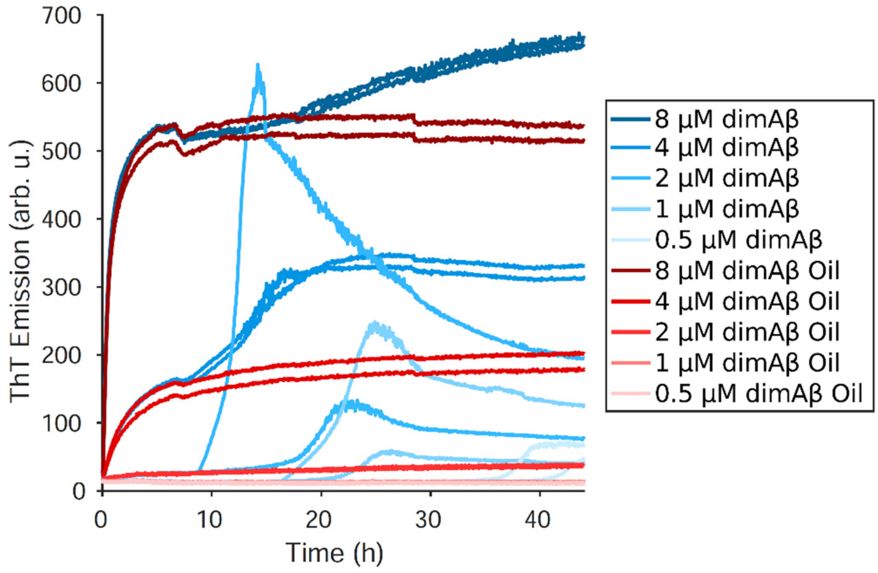

Supplementary Fig. 4 The air-water interface is crucial for fibril nucleation *in vitro*. Time courses of dimAβ assembly at pH 7.4 monitored by ThT fluorescence in a platereader. Half of the samples were covered by layering 10 µl of mineral oil on top of the aqueous solution. AβO formation was not impaired by mineral oil. Fibril nucleation, on the other hand, was retarded and not detectable during the whole timespan of the experiment.

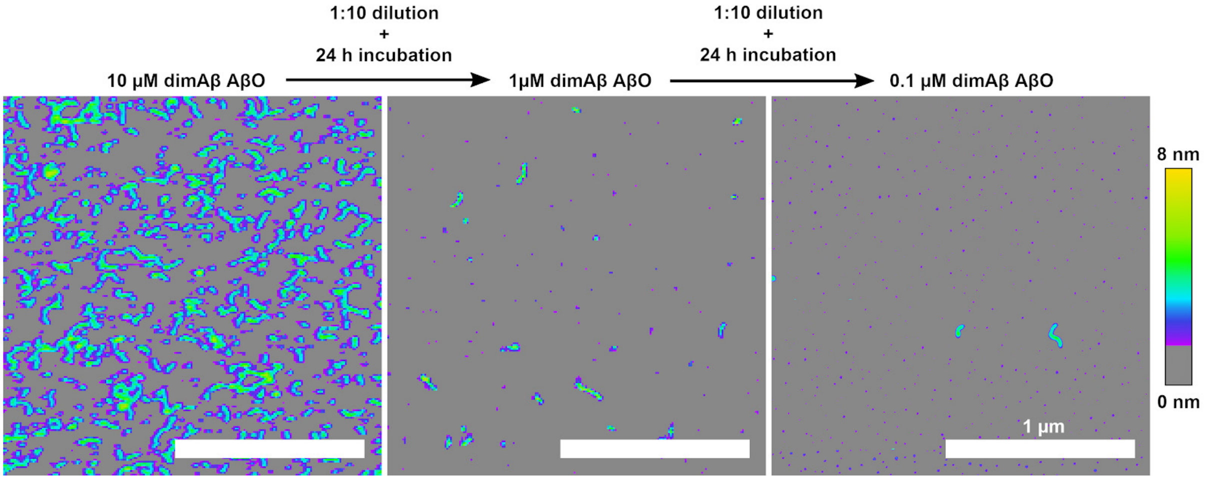

Supplementary Fig. 5 Long-term stability of diluted AβO. 10 µM dimAβ were quiescently incubated at 37°C, pH 7.4, for 72 h (left). Subsequently, the solution was diluted ten-fold to a dimAβ concentration of 1 µM and further quiescently incubated for 24 h at 37°C (middle). This solution was then further diluted ten-fold to a dimAβ concentration of 0.1 µM, which is far below the COC, and further incubated quiescently for 24 h at 37°C (right). Scalebar, 1 µm. *N*=1, between two and five micrographs were recorded for each condition.

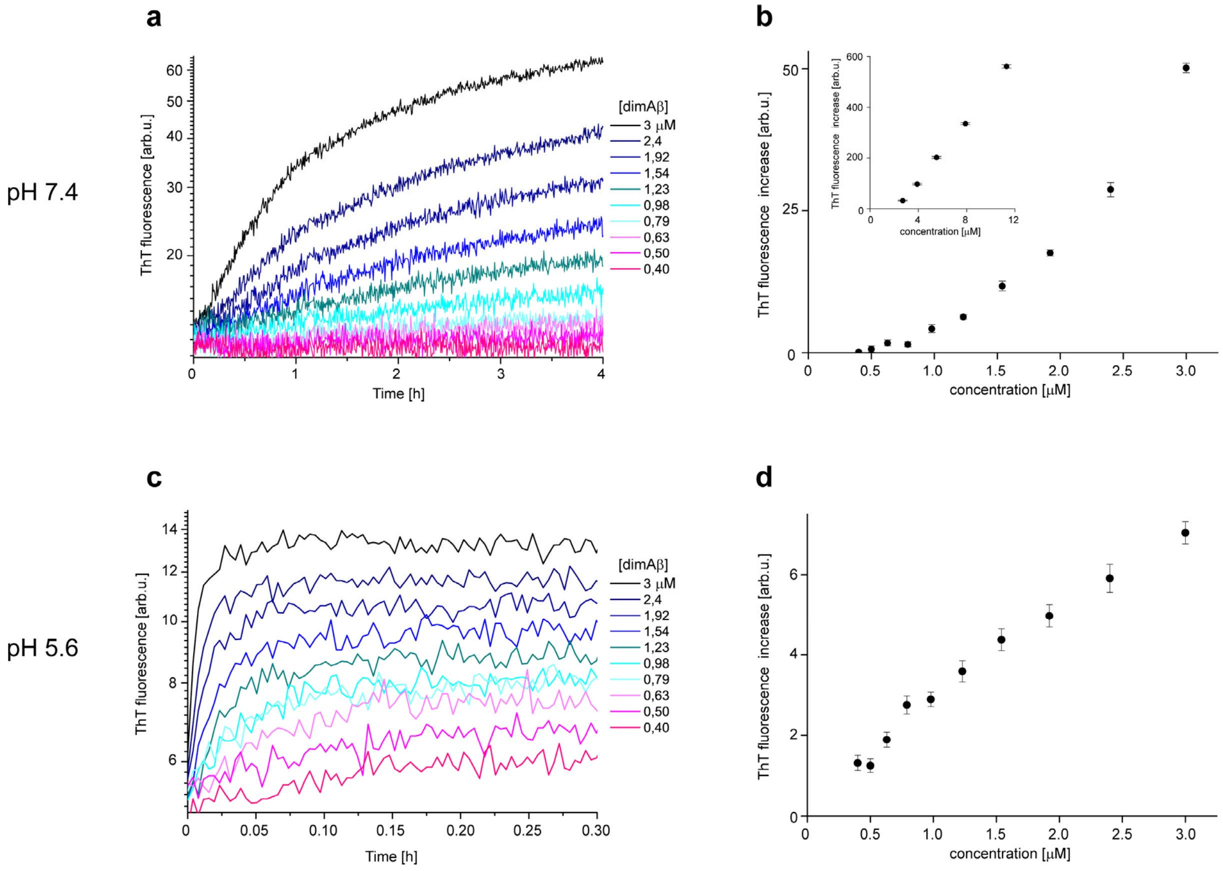

Supplementary Fig. 6 The critical concentration of AβO formation is reduced at acidic pH. Analysis of the AβO formation phase of dimAβ assembly at pH 7.4 (**a**, **b**) and pH 5.6 (**c**, **d**). **a**, **c** ThT fluorescence time traces at the indicated dimAβ concentrations. **b**, **d** The increase in ThT fluorescence intensity during the first 4 h (**b**) or 0.3 h (**d**) plotted against the dimAβ concentration. The inset in **b** shows data of a separate experiment spanning a range of higher concentrations. The data values in **b** and **d** are from single kinetic runs per concentration, with error bars representing the standard deviation of the final ten fluorescence readings in **a** and **c**. At pH 7.4, the intensity increase during the lag-free oligomerization phase scales linearly with protein concentration at dimAβ concentrations above ~2 μM (**b**, inset), whereas no lag-free oligomerization is detectable below ~0.5 μM, indicative of a COC of around 1 μM (**a**, **b**). At pH 5.6, however, there is no indication of disappearance of the oligomerization phase down to a concentration of 0.4 μM dimAβ (**c**, **d**). Due to the limited sensitivity of ThT at acidic pH it is not possible to reliably monitor oligomerization at lower concentrations and to determine the COC at this pH. Nevertheless, the COC at pH 5.6 is clearly lower than the COC at neutral pH, indicative of thermodynamic stabilization of AβOs at acidic pH.

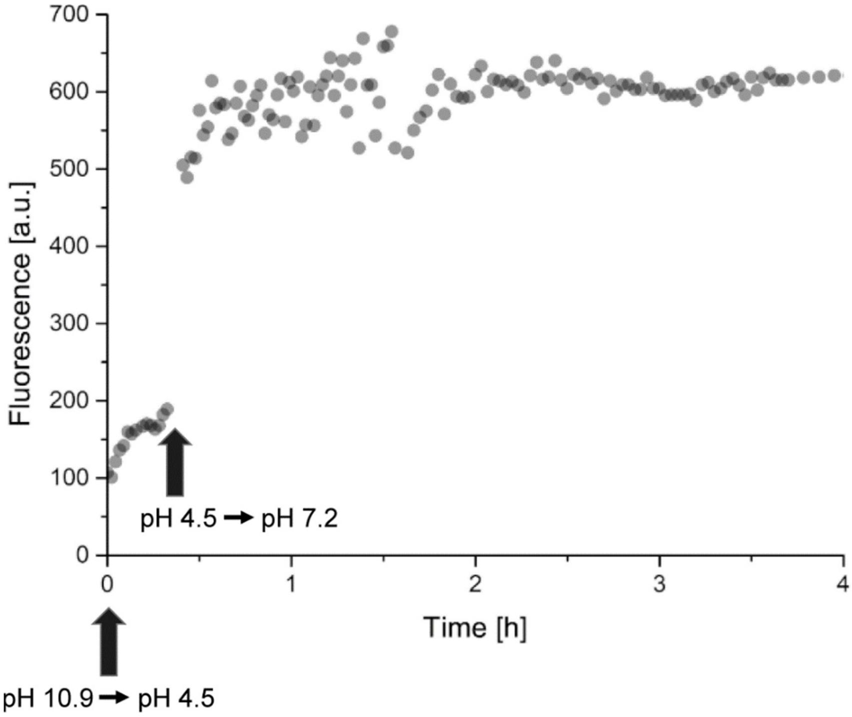

Supplementary Fig. 7 Stability of AβOs formed from Aβ42 at endo-lysosomal pH after shifting to neutral pH. ThT time course of AβO formation, initiated by pH adjustment from 10.9 to 4.5. Upon pH adjustment from pH 4.5 to pH 7.2 an immediate increase in fluorescence intensity is observed due to the pH sensitivity of ThT fluorescence. Apart from that, no other larger signal changes that would be expected in the case of disassembly of AβOs or replacement of AβOs by an alternative type of aggregate were observed.

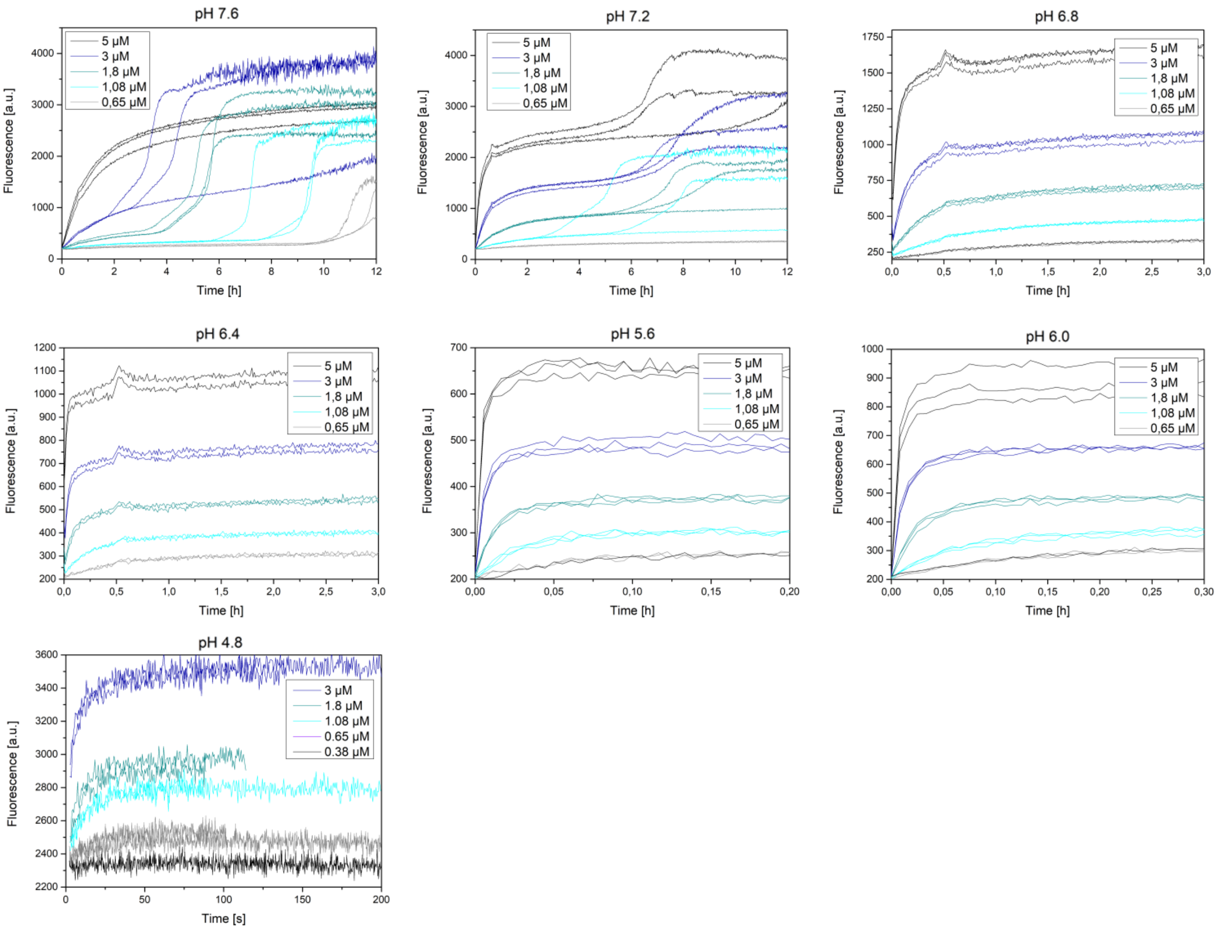

Supplementary Fig. 8 Replicate time traces from the dimAβ assembly experiment of Fig. 6.

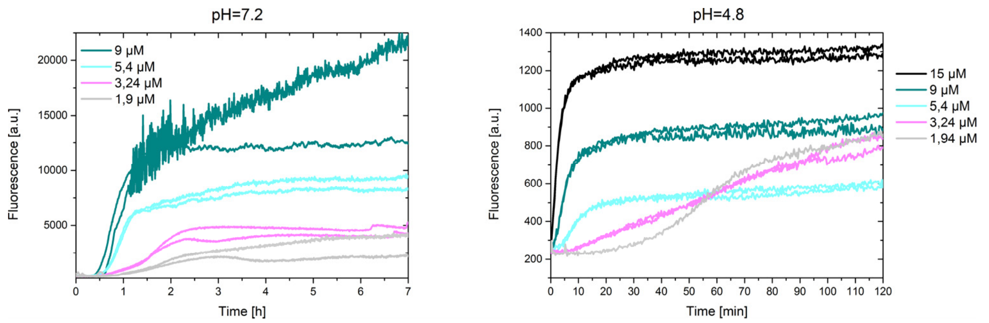

Supplementary Fig. 9 Replicate time traces from the Aβ42 assembly experiment of Fig. 7.

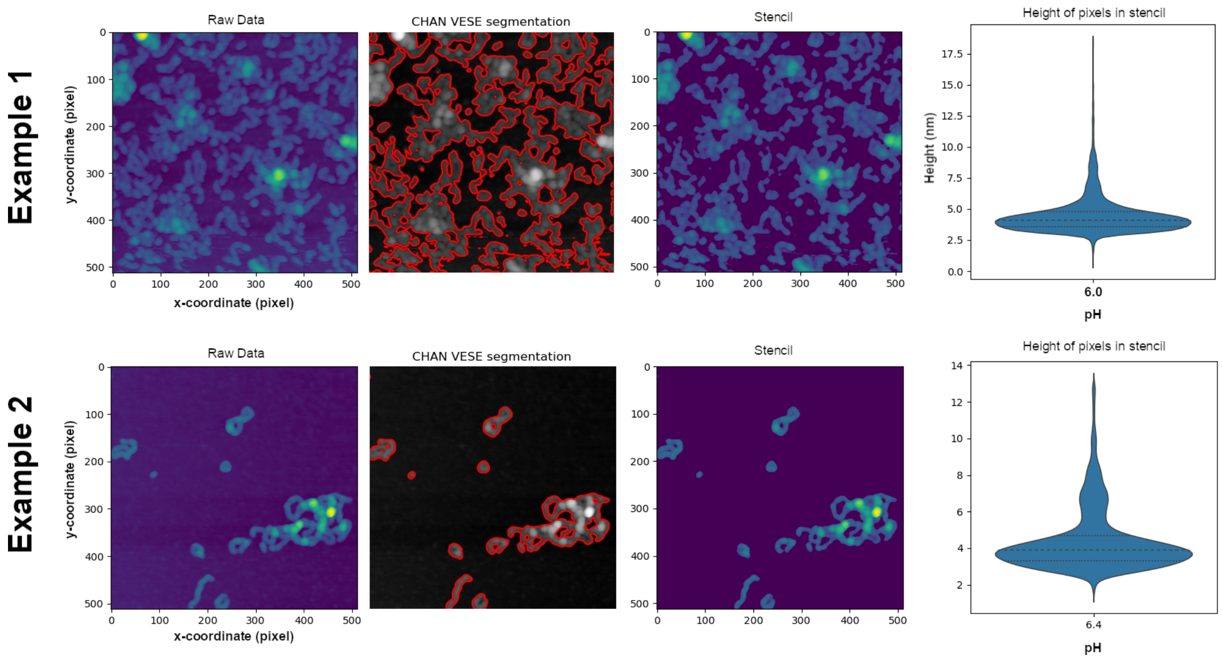

Supplementary Fig. 10 Examples of AFM image segmentation for height anaylsis. The Morphological Active Contours without Edges (MorphACWE) function 'morphological_chan_vese' of python's scikit-image module was used to distinguish and separate AβOs from background (typical settings were: iterations = 35; smooth = 1; lambda1 = 0.9; lambda2 = 0.89 to 0.895). Histographical height profiles of AβOs at different pH were determined as per pixel heights of the MorphACWE-isolated areas.
