# Supplementary material for: Endo-lysosomal Aβ concentration and pH trigger formation of Aβ oligomers that potently induce Tau missorting
Source: Nat Commun. 2021 Jul 30;12:4634. doi: 10.1038/s41467-021-24900-4 (PMC8324842; doi:10.1038/s41467-021-24900-4)
Supplement: Supplementary file 3 — Reporting summary [file 41467_2021_24900_MOESM3_ESM.pdf]

nature research

Corresponding author(s): Wolfgang Hoyer and Hans Zempel

Last updated by author(s): Jun 29, 2021

Reporting Summary

Nature Research wishes to improve the reproducibility of the work that we publish. This form provides structure for consistency and transparency in reporting. For further information on Nature Research policies, see our Editorial Policies and the Editorial Policy Checklist.

Statistics

For all statistical analyses, confirm that the following items are present in the figure legend, table legend, main text, or Methods section.

n/a	Confirmed	
☐	☒	The exact sample size (n) for each experimental group/condition, given as a discrete number and unit of measurement
☐	☒	A statement on whether measurements were taken from distinct samples or whether the same sample was measured repeatedly
☐	☒	The statistical test(s) used AND whether they are one- or two-sided<br>*Only common tests should be described solely by name; describe more complex techniques in the Methods section.*
☒	☐	A description of all covariates tested
☐	☒	A description of any assumptions or corrections, such as tests of normality and adjustment for multiple comparisons
☐	☒	A full description of the statistical parameters including central tendency (e.g. means) or other basic estimates (e.g. regression coefficient) AND variation (e.g. standard deviation) or associated estimates of uncertainty (e.g. confidence intervals)
☐	☒	For null hypothesis testing, the test statistic (e.g. F, t, r) with confidence intervals, effect sizes, degrees of freedom and P value noted<br>*Give P values as exact values whenever suitable.*
☒	☐	For Bayesian analysis, information on the choice of priors and Markov chain Monte Carlo settings
☒	☐	For hierarchical and complex designs, identification of the appropriate level for tests and full reporting of outcomes
☒	☐	Estimates of effect sizes (e.g. Cohen's d, Pearson's r), indicating how they were calculated

Our web collection on statistics for biologists contains articles on many of the points above.

Software and code

Policy information about availability of computer code

Data collection	BMG Reader Control (5.40), JPK Data Processing (spm-5.0.84), Leica LAS AF (3.2.), Leica LAS X (3.7.3), ZenBlue Pro imaging software (V2.5)
Data analysis	Origin (9.0), JPK Data Processing (spm-5.0.84), ImageJ (1.52p), GraphPad Prism v6, SPHIRE-crYOLO (1.3), CTFFIND4, Relion (3.0.5), VISDEM, DireX (0.7.1), Python (3.8.6) and module scikit image (0.17.2)

For manuscripts utilizing custom algorithms or software that are central to the research but not yet described in published literature, software must be made available to editors and reviewers. We strongly encourage code deposition in a community repository (e.g. GitHub). See the Nature Research guidelines for submitting code & software for further information.

Data

Policy information about availability of data

All manuscripts must include a data availability statement. This statement should provide the following information, where applicable:

- Accession codes, unique identifiers, or web links for publicly available datasets
- A list of figures that have associated raw data
- A description of any restrictions on data availability

The cryo-EM density map of dimAβ AβOs have been deposited in the Electron Microscopy Data Bank under accession code EMD-11327. Source data are provided with this paper. The authors declare that all the data necessary to interpret, verify and extend the research in the article are available within the article (and Supplementary Information files). All data are available from the corresponding authors on reasonable request.

Field-specific reporting

Please select the one below that is the best fit for your research. If you are not sure, read the appropriate sections before making your selection.

☒ Life sciences ☐ Behavioural & social sciences ☐ Ecological, evolutionary & environmental sciences

For a reference copy of the document with all sections, see nature.com/documents/nr-reporting-summary-flat.pdf

Life sciences study design

All studies must disclose on these points even when the disclosure is negative.

Sample size	No statistical methods were used to predetermine the sample size. Sample sizes for the experiments were chosen based on literature analysis and on previous experience with similar setups that showed significance (e.g. Zempel et al 2010, Hasecke et al 2018). Sample size is described for each experiment in the corresponding figure legend and additionally in the methods section.
Data exclusions	No data was excluded from this study.
Replication	ThT aggregation kinetics assays were repeated for #m independently prepared dimAβ/Aβ42 samples. In some of these assays, multiple repeats (separately pipetted multiplate wells) of concentration-dependent data sets were recorded, resulting in a total of #n data sets from #m indepedenty prepared samples, with #n/#m being: 6/3 (Fig. 1b), 6/2 (Fig. 6a), 6/2 (Fig. 6b), 6/2 (Fig. 6c), 5/4 (Fig. 6d), 8/4 (Fig. 6e), 6/4 (Fig. 6f), 2/2 (Fig. 6g), 6/2 (Fig. 7a), 6/2 (Fig. 7b). The repeats of the ThT assays of Fig. 6 and Fig. 7 are shown in Supplementary Figs. 8 and 9, respectively. AFM images were obtained for several areas on the substrate to ensure that the presented assembly types are representative for the respective sample. To determine particle height distributions in Fig. 6p, all pixels assigned to AβOs by the image analysis software in five micrographs per pH value were evaluated. Imaging of neuroblastoma cells was performed for three independently prepared samples on two different microscopes (for dimAβ) or for one cell culture preparation at three different Aβ concentrations (for Aβ42). All attempts to replicate the data were successful. Primary neuron cultures were seeded from 2-3 forebrains of different animals. For each replicate, independent cultures from different animals were used.
Randomization	Allocation of animals used in this study was random. Cells were randomly assigned to different experimental conditions after passing internal quality control measures.
Blinding	No blinding was performed during data acquisition, because acquisition was performed after standard procedures in an unbiased manner. Treatment analyses were performed in an unbiased manner, with pre-established criteria, and by different persons than those who prepared the samples.

Reporting for specific materials, systems and methods

We require information from authors about some types of materials, experimental systems and methods used in many studies. Here, indicate whether each material, system or method listed is relevant to your study. If you are not sure if a list item applies to your research, read the appropriate section before selecting a response.

Materials & experimental systems

n/a	Involved in the study	
☐	☒	Antibodies
☐	☒	Eukaryotic cell lines
☒	☐	Palaeontology and archaeology
☐	☒	Animals and other organisms
☒	☐	Human research participants
☒	☐	Clinical data
☒	☐	Dual use research of concern

Methods

n/a	Involved in the study	
☒	☐	ChIP-seq
☒	☐	Flow cytometry
☒	☐	MRI-based neuroimaging

Antibodies

Antibodies used	monoclonal mouse anti-Abeta [clone 4G8, Merck, #MAB1561], dilution: 1:300;<br>polyclonal rabbit anti-Tau [K9JA, Dako, A0024], dilution 1:1000
Validation	Tau antibody: WB of Sf9 cell lysates transfected with hTau cDNA; IHC, the antibody cross-reacts with the tau-equivalent protein in mouse and rat.<br>Abeta antibody: WB of Human Alzheimer disease brain lysate; IHC of paraffin-embedded Alzheimer's diseased hippocampus tissue

Eukaryotic cell lines

Policy information about cell lines

Cell line source(s)	SH-SY5Y cells were purchased from DSMZ-German Collection of Microorganisms and Cell Cultures (ACC 209).
Authentication	Authentication was performed by multiplex PCR of minisatellite markers.
Mycoplasma contamination	The cell line was tested negative for mycoplasma contamination.
Commonly misidentified lines (See ICLAC register)	No commonly misidentified lines were used in this study.

Animals and other organisms

Policy information about studies involving animals; ARRIVE guidelines recommended for reporting animal research

Laboratory animals	WT FVB/NJ mice E13.5 were obtained from CMMC animal facility or CECAD in vivo research facility (both Cologne, Germany). All Mice were housed in temperature (22°C) and humidity (40-60%) controlled rooms with a 12-hour light/dark cycle and fed standard mouse chow diet.
Wild animals	This study does not involve wild animals.
Field-collected samples	This study does not involve field-collected samples.
Ethics oversight	Since no experiments were carried out in living animals, no ethical approval was required. For the isolation of primary neurons a report (§4 TschG) for euthanasia of WT animals at the age E13.5 was reviewed and approved by the Animal Welfare Officer of the University of Cologne and by the Landesamt für Natur-, Umwelt- und Verbraucherschutz (LANUV), Germany.

Note that full information on the approval of the study protocol must also be provided in the manuscript.
